# The SCO4117 ECF Sigma Factor Pleiotropically Controls Secondary Metabolism and Morphogenesis in *Streptomyces coelicolor*

**DOI:** 10.3389/fmicb.2018.00312

**Published:** 2018-02-21

**Authors:** María T. López-García, Paula Yagüe, Nathaly González-Quiñónez, Beatriz Rioseras, Angel Manteca

**Affiliations:** Área de Microbiología, Departamento de Biología Funcional e IUOPA, Facultad de Medicina, Universidad de Oviedo, Oviedo, Spain

**Keywords:** *Streptomyces*, ECF, sigma factor, differentiation, secondary metabolism, antibiotic

## Abstract

Extracytoplasmic function (ECF) sigma factors are a major type of bacterial signal-transducers whose biological functions remain poorly characterized in streptomycetes. In this work we studied SCO4117, a conserved ECF sigma factor from the ECF52 family overexpressed during substrate and aerial mycelium stages. The ECF52 sigma factors harbor, in addition to the ECF sigma factor domain, a zinc finger domain, a transmembrane region, a proline-rich C-terminal extension, and a carbohydrate-binding domain. This class of ECF sigma factors is exclusive to Actinobacteria. We demonstrate that *SCO4117* is an activator of secondary metabolism, aerial mycelium differentiation, and sporulation, in all the culture media (sucrose-free R5A, GYM, MM, and SFM) analyzed. Aerial mycelium formation and sporulation are delayed in a *SCO4117* knockout strain. Actinorhodin production is delayed and calcium-dependent antibiotic production is diminished, in the Δ*SCO4117* mutant. By contast, undecylprodigiosin production do not show significant variations. The expression of genes encoding secondary metabolism pathways (deoxysugar synthases, actinorhodin biosynthetic genes) and genes involved in differentiation (*rdl, chp, nepA, ssgB*) was dramatically reduced (up to 300-fold) in the *SCO4117* knockout. A putative motif bound, with the consensus “CSGYN-17bps-SRHA” sequence, was identified in the promoter region of 29 genes showing affected transcription in the *SCO4117* mutant, including one of the *SCO4117* promoters. *SCO4117* is a conserved gene with complex regulation at the transcriptional and post-translational levels and the first member of the ECF52 family characterized.

## Introduction

Extracytoplasmic function (ECF) sigma factors, together with one- and two-component systems, are a major type of bacterial signal-transducing proteins (Huang et al., 2015). The ECF sigma factors belong to the σ^70^ family, but harbor only two of the four conserved regions of this group (σ^2^ and σ^4^ regions), which is sufficient for promoter recognition and RNA polymerase recruitment (reviewed in Mascher, 2013). The ECF sigma factors activate genes that confer resistance to agents that threaten the integrity of the envelope or cellular homeostasis (Kormanec et al., 2016) and are tightly regulated by diverse and complex mechanisms (Mascher, 2013). Most ECF sigma factors are negatively regulated by anti-σ factors (ASF), usually co-expressed with its target ECF sigma factor. The correct stimulus leads to the inactivation of the ASF and allows the ECF sigma factors to bind to their target promoters and RNA polymerase (Mascher, 2013). However, several ECF sigma factors lack a known ASF and are therefore differently regulated (Staron et al., 2009; Huang et al., 2015). Members of the ECF41 and ECF42 families possess C-terminal extensions that regulate their activities, thereby acting like ASFs (Gómez-Santos et al., 2011; Wecke et al., 2012), while the activity of other ECF sigma factors was proposed to be regulated by ASF-independent transcriptional regulators and post-translational modifications (Ser/Thr/Tyr phosphorylation) (Mascher, 2013).

*Streptomyces* is a genus of Gram-positive soil bacteria of great importance for biotechnology given their ability to produce a large array of bioactive compounds, including antibiotics, anticancer agents, immunosuppressants, and industrial enzymes (Hopwood, 2007). *Streptomyces* has a complex morphogenesis that includes hyphal differentiation and sporulation. In high density laboratory cultures, after spore germination, a fully compartmentalized mycelium (MI) initiates the development until it undergoes an ordered process of programmed cell death (PCD) and develops into a second multinucleated mycelium (substrate mycelium, early MII). This mycelium further develops into aerial hyphae and makes the hydrophobic proteins necessary for growth into the air (reviewed in Yagüe et al., 2013a). Secondary metabolism and differentiation are largely controlled by specific sigma factors that enable the recognition of specific promoters, directing the expression of specific genes (reviewed in Kormanec et al., 2016). *Streptomyces coelicolor*, the best-characterized *Streptomyces* strain, harbors 65 σ factors, including principal σ factors (*hrdA-D*), general stress response σ factors, and ECF sigma factors (Kormanec et al., 2016). The *S. coelicolor* genome encodes for 51 ECF sigma factors, of which only four have been characterized so far: SigE, required for a normal cell wall structure (Hutchings et al., 2006); SigR, a global regulator of redox homeostasis (Feeney et al., 2017); SigT, regulating actinorhodin production in response to nitrogen stress (Feng et al., 2011); and σBldN, an ECF sigma factor required for aerial mycelium formation (Bibb et al., 2012).

**Figure 1:**
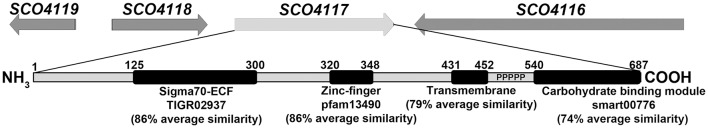
*SCO4117* structure. Outline of the *SCO4116*-*SCO4119* region, showing the SCO4117 conserved domains and average similarities between *SCO4117* and their orthologs in *S. griseus, S. avermitillis, S. lividans, S. clavuligerus*, and *S. venezuelae*. Ps indicate the proline-rich C-terminal extension.

The ECF52 family of sigma factors is characterized by long C-terminal extensions that contain a zinc finger domain, a variable number of transmembrane helices and a long proline-rich C-terminal extension, which includes a carbohydrate-binding domain (Huang et al., 2015; Figure 1). Members of this family are only present in Actinobacteria (Huang et al., 2015). In this work, we studied *SCO4117*, a conserved ECF52 sigma factor that was detected as overexpressed during the substrate and aerial mycelia stages of *Streptomyces* development (Yagüe et al., 2013b), suggesting a role in the regulation of secondary metabolism and differentiation. To our knowledge, *SCO4117* is the first member of the ECF52 sigma factors that has been characterized.

## Materials and methods

### Bacterial strains and culture conditions

All *Streptomyces* and *Escherichia coli* strains used in this work are listed in Table 1. Spores were harvested from SFM solid plates (Kieser, 2000) after growth at 30°C for 7 days. The differentiation analyses were carried out on GYM plates covered with cellophane and on minimal medium (MM) plates supplemented with glucose (10 g/l), both inoculated with 10^7^ spores from a fresh water suspension and cultured at 30°C. The samples for quantification of actinorhodin and undecilprodigiosin production were obtained from 100-ml sucrose-free R5A (Fernández et al., 1998) cultures grown at 30°C and 200 rpm in 500-ml flasks. Calcium-dependent antibiotic (CDA) production was measured on nutritive agar from Oxoid. *E. coli* strains were cultured in LB and 2xTY media at 37°C. The following antibiotics were added to select plasmid-bearing and mutant strains: ampicillin (100 μg/ml), apramycin (100 μg/ml *for E. coli*, 25 μg/ml for *S. coelicolor*), chloramphenicol (25 μg/ml), hygromycin (100 μg/ml *for E. coli*, 200 μg/ml for *S. coelicolor*), kanamycin (50 μg/ml), and nalidixic acid (25 μg/ml).

**Table 1:** Bacterial strains, plasmids, cosmids, and primers used in this study.

**Strain, plasmid, cosmid**	**Description**	**Reference**
*S.coelicolor* M145	SCP1^−^ SCP2^−^, reference strain	Kieser, 2000
S.*coelicolor ΔSCO4117*	*SCO4117* replaced with *acc(3)IV*, Apr^R^	This study
*E. coli* TOP10	F^−^*mcr*A Δ(*mrr*-*hsd*RMS-*mcr*BC) ϕ80*lac*ZΔM15 Δ*lac*X74 *rec*A1 *ara*D139 Δ(*ara*-*leu*)7697 *gal*U *gal*K *rps*L *end*A1 *nup*G	Invitrogen
*E. coli* ET12567	*dam-13::*Tn9*, dcm-6, hsdM, hsdR*	MacNeil et al., 1992
*E. coli* ET12567/pUZ8002	*E. coli* ET12567 harboring pUZ8002, a not self-transmissible plasmid which can mobilize *oriT*-containing plasmids by conjugation	Flett et al., 1997
*Bacillus subitilis*	Indicator microorganism for CDA bioassay	
**PLASMIDS AND COSMIDS**		
pMS82	Integrative and conjugative vector, Hyg^R^	Gregory et al., 2003
PCR™-Blunt II-TOPO®	Zero Blunt® TOPO® PCR Cloning Kit, Kan^R^	Invitrogen
pIJ773	Apr cassette in pIJ699	Gust et al., 2003
pΔ*SCO4117*	*SCO4117* deletion construction, a *Streptomyces* non-replicative plasmid transmissible by conjugation, Apr^R^ Kan^R^	This study
pMS82-*SCO4117*	Integrative and conjugative plasmid derived from pMS82 with the completed SCO4117 gen under its own promoter control. Hyg^R^	This study
pCRISPR-Cas9	Conjugative and thermosensitive plasmid harboring Cas9	Tong et al., 2015
pCRISPR-120	pCRISPR-Cas9 harboring the apramycin resistance target sequence	This study
pCRISPR-SCO4117	pCRISPR-120 harboring a 3.7 kb DNA fragment including SCO4117	This study
pCRISPR-SgSCO4117	pCRISPR-Cas9 harboring the target *SCO4117* sequence	This study
pCRISPR-SCO4117A	pCRISPR-SgSCO4117 harboring a 2.5 kb fragment used to create the *SCO4117* knockout	This study
pCRISPR-SCO4117B	pCRISPR-SgSCO4117 harboring a 2.2 kb fragment used to create the truncated *SCO4117* gene harboring the sigma factor domain	This study
**PRIMERS**		
SCO134	GACGTGCTGCTGGTCATAGC	This study
SCO4117R	GGGACTAGTGTACGCCGCCGAAGTGG	This study
SCO159	GGAGGCGATGTCCATCTGTT	This study
SCO160	TCAACGCTCATCGCGGAAAG	This study
SCO163	CTGCAGGCCCAGTCCCGCTTCAC	This study
SCO164	GATATCACTCCTGGTCCTCGACAACT	This study
SCO4848F	CGTCGTATCCCCTCGGTTG	Gonzalez-Quiñonez et al., 2016
pMS82R	GAGCCGGGAAAGCTCATTCA	Gonzalez-Quiñonez et al., 2016
SCO197	CATGCCATGGTTCCGCGATGAGCGTTGAAAGTTTTAGAGCTAGAAATAGC	This study
sgRNA-R	ACGCCTACGTAAAAAAAGCACCGACTCGGTGCC	Tong et al., 2015
SCO4117-FA	GGGGGATATCACGACCGTGATCTCGGCC	This study
RT-SCO4117-F	CGACGACACGGCCTACGA	This study
RT-SCO4117-R	CGCACCGCCTGAAGCAT	This study
hrdB-F	CGCGGCATGCTCTTCCT	Kurt et al., 2013
hrdB-R	AGGTGGCGTACGTGGAGAAC	Kurt et al., 2013
bldN-F	CTCACCAGCGAGACCTTTCTGC	This study
bldN-R	TCGTTGGCGTCGAGCATCT	This study
SCO1178-F	TCAAGGTCCGGCAGGTCTA	This study
SCO1178-R	CCGTCCTCCTGCTTGGT	This study
chpA-F	CTCGTCCTCGTCCTCGACTT	Straight et al., 2006
chpA-R	GTCGTTCTCGCACTTGTTGC	Straight et al., 2006
chpH-F	CACCGGTGGTCTGGTTCTC	Straight et al., 2006
chpH-R	ATCACGGAGATCGTGTTGC	Straight et al., 2006
SCO2748-F	GAGATCACCCCGAAACTGG	This study
SCO2748-R	AAGTGCCAGTCGATGACGTT	This study
actVA2-F	ACTACGCCTCCCAGAACCTC	This study
actVA2-R	TTGTGCCCGCCGATGTC	This study
redF-F	CGGAGAACAAGGGCAAGC	This study
redF-R	CAGGGGGATGGCGAAG	This study
actII4-F	GCGGCTTTTTGGAATGC	This study
actII4-R	GCAGGGTCTCGTTCAGC	This study
SCO215	CATGCCATGGCAGCTCGCTCCACTGGTAGAGTTTTAGAGCTAGAAATAGC	This study
SCO216	GATATCCGGGAACCAACGTCGCACGG	This study
SCO217	CGGTTCCGCAGGCTCCTACGCGGAAAGCCCCCGCCAAC	This study
SCO218	GTTGGCGGGGGCTTTCCGCGTAGGAGCCTGCGGAACCG	This study
SCO219	GATATCTGCAGGTGCGGATGATGCAC	This study
SCO220	GATATCCGTATCTCCTTCCCCGAACG	This study
SCO221	CGGTTCCGCAGGCTCCTACTGGAGGTAGGCCTGCTTG	This study
SCO222	CAAGCAGGCCTACCTCCAGTAGGAGCCTGCGGAACCG	This study
Mut4117F	CCCTGTCGCAACCTCTGC	This study
Mut4117R	GACGGGCACCCTGCG	This study

### Nucleic acid manipulations

Genomic DNA isolation and conjugation were performed following standard methods (Kieser, 2000; Sambrook and Russell, 2001).

Total RNA samples were isolated as previously described (Rioseras et al., 2016) using RNeasy Mini spin columns and treated with DNase I (Qiagen). The quantity and integrity of the RNA samples were measured with Nanodrop 2000 (Thermo Scientinfic) and 2100 Bioanalyzer (Agilent).

### *SCO4117* mutagenesis

The *SCO4117* ORF was replaced with the apramycin resistance cassette. The fragments upstream (UP-1944pb) and downstream (DW-2234pb) of *SCO4117* were amplified by PCR from *S. coelicolor* DNA using the primers SCO159-SCO160 and SCO163-SCO164, respectively. The PCR products were cloned and sequenced in pCR™-Blunt II-TOPO® obtaining pTOPO-UP and pTOPO-DW. The DW fragment was liberated from pTOPO-DW by *EcoR*V/*Pst*I digestion and subcloned in pTOPO-UP digested with the same restriction enzymes. The resulting plasmid, pTOPO-UPDW, was linearized with *Pst*I and ligated with a *Pst*I-fragment from pIJ773 containing an apramycin resistance cassette with an *oriT*, obtaining pΔ*SCO4117*. This vector was introduced in the wild-type strain by conjugation and double recombinants were selected as kanamycin-sensitive and apramycin-resistant. Southern hybridization and PCR amplification with SCO134 and 4117R (Table 1) primers were performed to check the Δ*SCO4117* mutant.

To discard polar effects in the expression of the *SCO4117* neighbor genes due to the apramycin gene insertion, we recreated the *SCO4117* knockout by CRISPR-Cas9. We used the system designed by Tong et al. (2015). The 20-nt target sequence was selected inside the *SCO4117* and amplified by PCR with the primers SCO215 and sgRNA-R. The product of 110 bps was digested with *Nco*I/*SnaB*I and cloned in *Nco*I/*SnaB*I-digested pCRISPR-Cas9, obtaining pCRISPR-SgSCO4117. The *SCO4117*-surrounding regions were amplified by PCR with SCO216/SCO217 and SCO218/SCO219 primers. The couple of DNA fragments were combined by overlap extension PCR (Lee et al., 2010) with the primers SCO216/SCO219. The PCR product was cloned and sequenced in pCR™-Blunt II-TOPO®. The insert was released with *EcoR*V and cloned into pCRISPR-SgSCO4117 digested with *Stu*I. The final vector pCRISPR-SCO4117A was introduced into the *S. coelicolor* wild-type strain by conjugation. The conjugants harboring the plasmid were selected using apramycin resistance, plated in SFM and grown at 37°C for 3 days for plasmid clearance. Mutations were confirmed by PCR using the primers Mut4117F and Mut4117R (Table 1).

The SCO4117 mutant harboring the nucleotides encoding for the first 305 amino acids of *SCO4117*, those including the sigma factor domain (Figure 1), was created by CRISPR-Cas9. The 20-nt target sequence was selected inside the *SCO4117*, but outside the first 305 amino acids (Figure 1) and amplified by PCR with the primers SCO215/sgRNA-R. The SCO220/SCO221 and SCO222/SCO219 primers were used (primer SCO222 contain the SCO4117 stop codon in frame with the first 305 amino acids) to amplify the *SCO4117* sigma factor-surrounding regions. DNA fragments were combined by overlap extension PCR with the primers SCO220/SCO219. The amplicon was cloned and sequenced in pCR™-Blunt II-TOPO®. The insert was released with *EcoR*V and cloned into pCRISPR-SgSCO4117 digested with *Stu*I, obtaining vector pCRISPR-SCO4117B. Conjugation, mutagenesis, and mutant confirmation was performed as described above (Table 1).

### *S. coelicolor* Δ*SCO4117* complementation

The complementation of *S. coelicolor* Δ*SCO4117* was performed via the integration of plasmid pMS82 (Gregory et al., 2003), harboring the *SCO4117* ORF and an upstream region large enough to include the two promoter regions identified by Jeong et al. (2016). The *SCO4117* was amplified from the *S. coelicolor* chromosome using the primers SCO134 and 4117R. The 2171 bp-fragment was cloned in pCR™-Blunt II-TOPO® and sequenced to check the absence of mutations. A *Spe*I fragment obtained from TOPO-4117 was ligated into pMS82 digested with *Spe*I resulting in pMS82-*SCO4117*. This plasmid was transferred by conjugation to the *S. coelicolor* Δ*SCO4117* mutant. The conjugants harboring the plasmid were selected by hygromycin resistance and the correct plasmid insertion at the *SCO4848 attB* site was verified by PCR using the primers SCO4848F and pMS82R (Table 1).

Restoration of the wild-type genotype of the Δ*SCO4117* mutant was achieved using the CRISPR-Cas9 system for *Streptomyces* designed by Tong et al. (2015). The 20-nt target sequence was selected inside the apramycin resistance cassette, replacing *SCO4117* and amplified by PCR with the primers SCO197 and sgRNA-R. The 120-bps product was digested with *Nco*I/*SnaB*I and cloned in *Nco*I/*SnaB*I-digested pCRISPR-Cas9 obtaining pCRISPR-120. A single fragment of 3.7 kb, containing the complete *SCO4117* sequence and the surrounding regions, was amplified by PCR from *S. coelicolor* chromosome using SCO4117-FA and SCO4117-R primers. The product was then cloned and sequenced in pCR™-Blunt II-TOPO®. The resulting plasmid was digested with *EcoR*V/*Spe*I to liberate a 3.7 kb fragment. The *Spe*I-end was filled with the Klenow enzyme and the product was cloned into pCRISPR-120 linearized with *Stu*I. The final vector pCRISPR-4117, was introduced in *S. coelicolor* Δ*SCO4117* by conjugation. Conjugant selection and plasmid clearance were performed as described above. Restoration of the wild-type genotype was checked by the loss of apramycin resistance (Table 1).

### Viability staining

Samples were obtained from GYM plates covered with cellophane at different developmental stages. The bacteria were stained with SYTO 9 and propidium iodide (LIVE/DEAD Bac- Light Bacterial Viability Kit, Invitrogen, L-13152) and observed under a Leica TCS-SP8 confocal laser-scanning microscope at wavelengths of 488 and 568 nm excitation and at 530 (green) or 640 nm (red) emissions.

### Antibiotic production and protein quantification

Undecylprodigiosin and actinorhodin were quantified spectrophotometrically according to Tsao et al. (1985) and Bystrykh et al. (1996). For actinorhodin quantification, KOH was added to the culture samples at a final concentration of 1N. Cellular pellets were discarded by centrifugation and actinorhodin concentration was spectrophotometrically determined at 640 nm applying the linear Beer–Lambert relationship (ε_640_ = 25,320). The culture samples for undecylprodigiosin quantification were vacuum-dried, resuspended in methanol, acidified with 0.5N HCl and spectrophotometrically assayed at 530 nm, using the Beer–Lambert relationship to estimate concentration (ε_530_ = 100,500).

CDA production was determined via a bioassay against *Bacillus subtillis*. Oxoid nutritive agar (ONA) plates (90 mm in diameter) were inoculated with 5 μl of a *Streptomyces* spore suspension at 1 × 10^5^ spores/ml and incubated at 30°C. After 2 days, the plates were overlayed with 5 ml of soft ONA (0.75% agar), inoculated with *B. subtilis* (OD = 0.25) and supplemented with Ca(NO_3_)_2_ (60 mM). Negative controls were performed in parallel without adding calcium. Inhibitory halos were measured after 15 h at 30°C.

Growth was determined by measuring the protein concentration with the Bradford assay (Biorad) and a bovine serum albumin standard (Sigma). Total protein extracts were obtained mixing a volume of culture with a volume of 1 M NaOH, boiling for 5 min, and removing cell debris by centrifugation at 7740 g.

### RNA-seq and bioinformatic analysis

Next-generation sequencing (NGS) was performed by Stab Vida (Caparica, Portugal) from two biological replicates using the Δ*SCO4117* mutant and the *S. coelicolor* wild-type strain. Ribosomal RNA was depleted with the Ribo-Zero Bacteria Kit (Illumina), and the cDNA library construction was carried out using the TruSeq Stranded mRNA Library Preparation Kit (Illumina). The DNA was sequenced in the Illumina HiSeq 2500 platform using 100-bp paired-end sequencing reads (at least 20 M reads per sample). Raw data are available via the Gene Expression Omnibus database (accession GSE107661).

Bioinformatic analysis of the sequenced data was performed under the Linux operative system using the following software: FastQC to check the quality of the sequences, Cutadapt for trimming sequences, Bowtie2 for mapping with the *Streptomyces coelicolor* genome and Cuffdiff for differential expression test analysis (Trapnell et al., 2012). Variations in transcript abundances were considered significant if they had a *p*-value < 0.03 (Supplementary Table 1). Transcript abundances without significant variations (*p*-values higher than 0.03) are shown in Supplementary Table 2.

The transmembrane topology of the *SCO4117* gene was analyzed using the Phobius software (http://phobius.sbc.su.se/). The *SCO4117* orthologs were obtained from the Strepdb database (http://strepdb.streptomyces.org.uk/): SLI_4349 (*S. lividans*), SAV_3491 (*S. avermitilis*), SVEN15_3779 (*S. venezuelae*), SGR_3904 (*S. griseus*), and SCLAV_3143/ SCLAV_3144 (*S. clavuligerus*). Amino acid similarities were estimated using the software package Lalign (http://www.ch.embnet.org/software/LALIGN_form.html).

### SCO4117 motif bound search

The 46 genes significantly down-regulated in the Δ*SCO4117* mutant were grouped into 33 putative operons (Supplementary Table 1). A library of putative regulatory sequences has compiled from the 250 nucleotide-long sequences located upstream of the putative operons. BioProspector (Liu et al., 2001) was used to search for bipartite overrepresented motifs in these sequences. Searches were performed only in the forward strand, and the following parameters were varied iteratively: the lengths of the −35 and −10 motifs were varied between 5 and 7 nucleotides; the spacer length was varied between 15 and 20 nucleotides in 1-nucleotide intervals. From all generated motifs, the highest scoring bipartite motif was selected.The motif logo was created using Weblogo (Crooks et al., 2004).

### Quantitative RT-PCR (qRT-PCR)

A High-Capacity cDNA Reverse Transcription Kit (Applied Biosystems) was used to synthetize cDNA from 0.5 μg of RNA from two biological replicates. Real-Time PCRs of the *SCO4117* gene were carried out on an ABI PRISM 7900 HT thermocycler (Applied Biosystems). The reactions were performed in triplicate, containing 2 μl of 2-fold diluted cDNA, 10 μl of SYBR Green PCR Master Mix (Applied Biosystems), and 300 nM of specific primers (RT-SCO4117-F/R) (listed in Table 1) in a final volume of 20 μl. The *hrdB* (SCO5820, amplified using primers hdrB-F/R) was used as reference since its expression showed no variation between strains in our RNA-seq results (Supplementary Table 2). The DNA contamination and primer dimer amplification were tested in negative controls replacing cDNA by RNA or water. Amplification conditions were as follows: 2 min at 50°C, 10 min at 90°C, 40 repetitions of 15 s at 95°C, and 1 min at 60°C. Primer efficiencies were measured using serial dilutions of genomic DNA as template and the relative quantification of gene expression was performed by the ΔΔCt method (Livak and Schmittgen, 2001).

The expression of the most differentially expressed genes between the *SCO4147* mutant and the wild strain was validated by qRT-PCR: *SCO5077, actVA2*; *SCO2478*, reductase activated by actinorhodin; and *SCO1178* epimerase. We also analyzed key genes from the actinorhodin (*actII-4, SCO5085*) and undecylprodigiosin (*redF, SCO5898*) clusters; one of the bald genes (*bldN, SCO3323*); two of the genes participating in the aerial hyphae formation (*chpA, SCO2716* / *chpH, SCO1675*); and *SCO0761*, a hypothetical protein (primers are listed in Table 1). The correlation between transcript abundances quantified by RNA-seq and qRT-PCR was adequate (regression coefficient of 0.71) (Supplementary Figure 1).

## Results

### *SCO4117* gene structure and conservation in the *Streptomyces* genus

Gene *SCO4117*, the only member of the ECF52 family present in *S. coelicolor*, encodes a conserved multidomain protein (average protein similarity of 68.4% among *S. griseus, S. avermitillis, S. lividans, S. clavuligerus*, and *S. venezuelae*). The *SCO4117* harbors a putative ECF sigma factor domain (conserved domain database accession TRIGR02937, 86% average similarity), a putative zinc finger domain (pfam13490, 86% average similarity), a putative transmembrane domain (79% average similarity), a putative carbohydrate-binding module (smart00776, 74% average similarity), and a proline-rich region separating the transmembrane and the carbohydrate module (Figure 1). Surprisingly, in the case of the *S. clavuligerus* ortholog, there is a stop codon separating the carbohydrate-binding domain module ORF (*SCLAV_3143*) from the other domain ORFs (*SCLAV_3144*) (http://strepdb.streptomyces.org.uk/), perhaps due to an error in the sequence at the proline-rich region. Gene *SCO4117* is localized downstream the actinorhodin positive regulator *atrA* (*SCO4118*), but both genes were described as being expressed independently (Uguru et al., 2005). This synteny is maintained in all *Streptomyces* species analyzed, with the exception of *Streptomyces avermitilis*, in which the *SCO4117* and *atrA* orthologs (*SAV_3491* and *SAV_4110* respectively) are separated.

### Mutation of *SCO4117* affects antibiotic production and morphogenesis in different culture conditions (liquid and solid) and media (sucrose-free R5A, GYM, MM, SFM)

**Figure 2:**
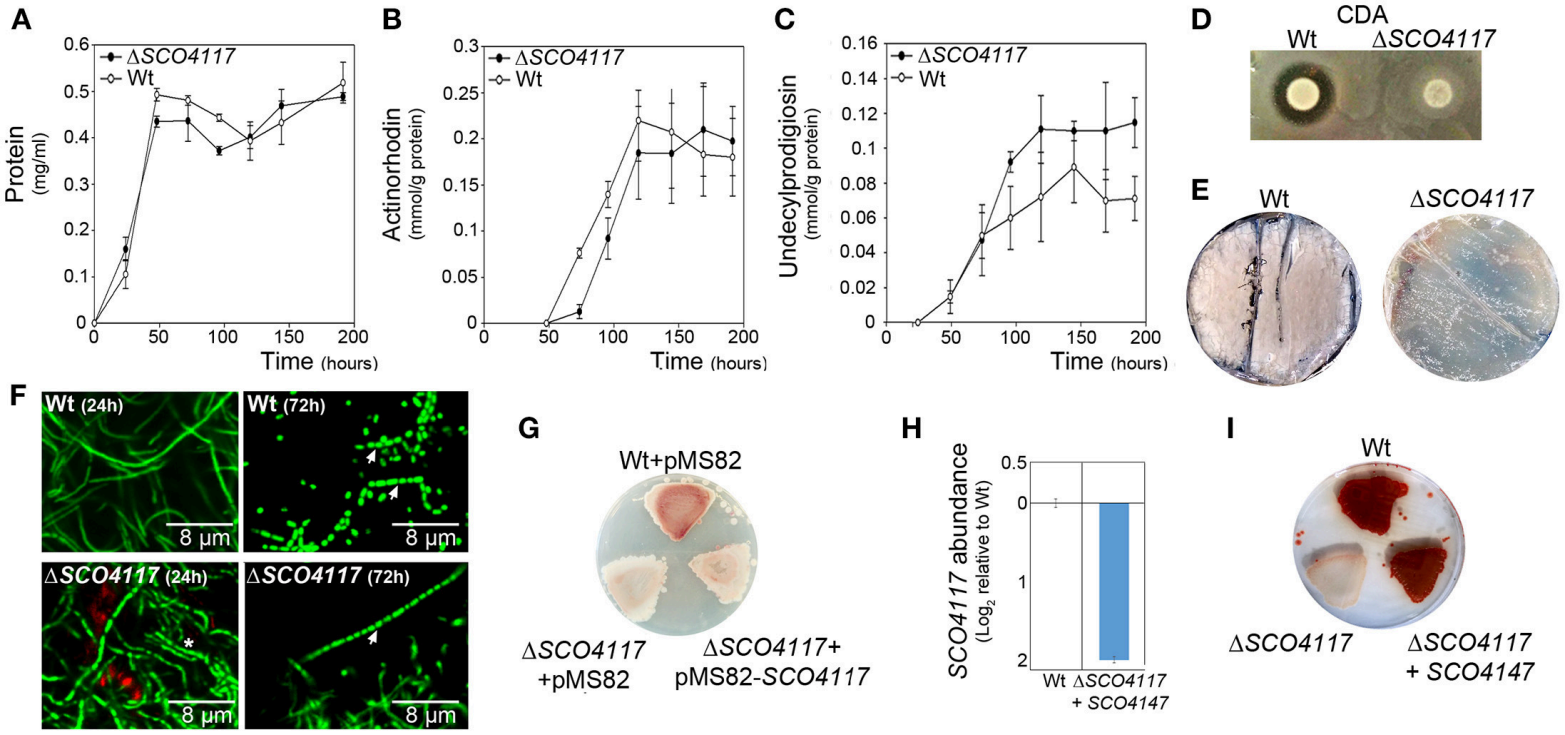
Phenotypical analyses of the Δ*SCO4117* mutant. **(A)** Growth curve. **(B)** Actinorhodin production. **(C)** Undecylprodigiosin production. **(D)** CDA production. **(E)** Macroscopic view of sporulation (gray color) in the Δ*SCO4117* mutant compared to the wild strain in GYM plates at 85 h. **(F)** Confocal laser fluorescence microscopy pictures (SYTO9-PI staining) of the Δ*SCO4117* mutant illustrating delay in MII differentiation (24 h) and sporulation (72 h) compared to the *S. coelicolor* M145 wild-type strain (GYM plates). Arrows indicate spore chains; the asterisk indicates the discontinuities characterizing the MI compartmentalized hyphae. **(G)** Macroscopic view of antibiotic production (red color) in the wild-type strain, the Δ*SCO4117* mutant harboring plasmid pMS82 and the Δ*SCO4117* mutant harboring plasmid pMS82-*SCO4117*, all of them grown in MM plates at 5 days. **(H)** *SCO4117* transcript abundance in the Δ*SCO4117* mutant harboring pMS82-SCO4117 (complemented mutant) compared to the wild-type strain in GYM plates at 17 h. **(I)** Macroscopic view of antibiotic production (red color) in the Δ*SCO4117* restored mutant in MM plates at 5 days.

The *S. coelicolor SCO4117* knockout mutant (Δ*SCO4117*) do not show a significant variation in growth (Figure 2A), or in the maximum amount of actinorhodin and undecylprodigiosin production (Figures 2B,C), in sucrose-free R5A liquid cultures. However, the mutant shows a delay in actinorhodin production compared to the wild-type strain (Figure 2B). The *SCO4117* knockout mutant showed lower CDA production (Figure 2D) and a delay in MII differentiation (notice the discontinuities characterizing the MI hyphae in the mutant at 24 h) (Figure 2F) (Manteca et al., 2006) and sporulation (Figures 2E,F) in solid GYM solid cultures. The delay in development was not complemented when *SCO4117* and an upstream region large enough to include the two promoter regions identified by Jeong et al. (2016) were introduced into the mutant using the integrative plasmid pMS82-*SCO4117* (Figure 2G). As discussed below, the *SCO4117* gene expression was not restored in the complemented mutant (the *SCO4117* transcript abundance was 4-fold less than in the wild-type strain) (Figure 2H). Interestingly, the Δ*SCO4117* phenotype was only restored when a copy of the *SCO4117* ORF was introduced at the native position in the Δ*SCO4117* mutant, using the CRISPR-Cas9 methodology (Figure 2I). This result discards the possibility of artifactual mutations at chromosomal positions different to *SCO4117*, generating the phenotypes observed.

**Figure 3:**
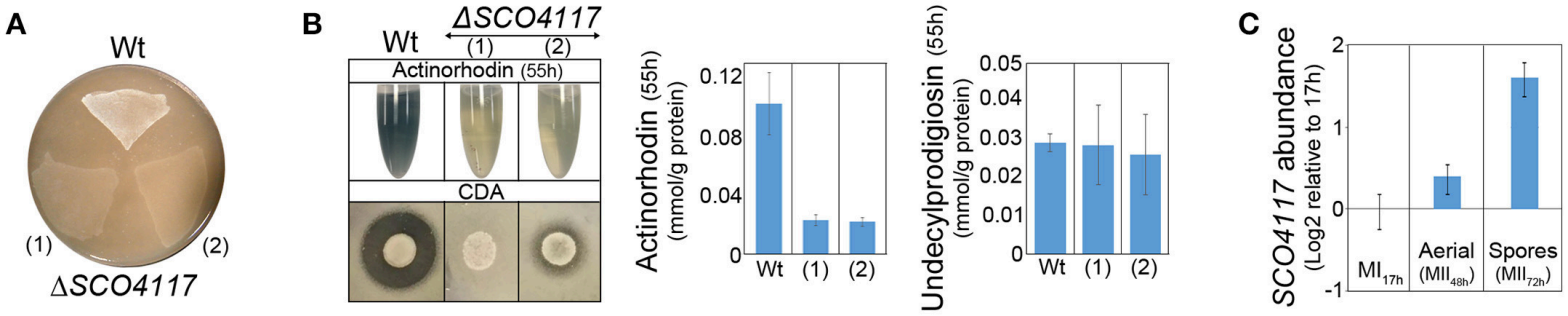
Phenotypical analyses of the CRISPR/Cas9-Δ*SCO4117* mutant and *SCO4117* gene expression. **(A)** Macroscopic view of aerial mycelium development (white color) in the Δ*SCO4117* mutant (1) and in the CRISPR/Cas9-Δ*SCO4117* mutant (2), both grown in SFM plates at 2 days. **(B)** Actinorhodin (sucrose-free R5A extracellular medium, purple color), CDA and undecylprodigiosin production in the Δ*SCO4117* and CRISPR/Cas9-Δ*SCO4117* mutants compared to the wild-type strain. **(C)** *SCO4117* transcript abundance at 17, 48, and 72 h in the *S. coelicolor* wild-type strain.

As discussed below, the above data suggest a complex regulation of the expression of the *SCO4117* gene. Polar effects in the expression of *SCO4117* neighbor genes due to the apramycin-resistance gene transcription, are unlikely, because, as detailed below, we did not detect significant variations in the expression of the *SCO4116* and *SCO4118* genes in the Δ*SCO4117* mutant compared to the wild-type strain (Supplementary Table 2). In order to further discard a possible effect of the apramycin-resistance gene in the phenotypes observed, we recreated the knockout mutant by CRISPR/Cas9. We eliminated the *SCO4117* ORF, obtaining the CRISPR/Cas9-Δ*SCO4117* mutant (strain “1” in Figures 3A,B). This mutant, shows a phenotype similar to the Δ*SCO4117* mutant (strain “2” in Figures 3A,B).

### *SCO4117* gene expression during *S. coelicolor* development

The expression of *SCO4117* was analyzed at three key developmental stages (MI, 17 h; aerial mycelia, 48 h; sporulating mycelia, 72 h) by qRT-PCR, in solid GYM cultures of the wild-type strain. As reported previously in our transcriptomic work (Yagüe et al., 2013b), *SCO4117* is overexpressed during the sporulating stage in the *S. coeliolor* wild-type strain (Figure 3C).

### The SCO4117 sigma factor regulates the expression of its own gene

**Figure 4:**
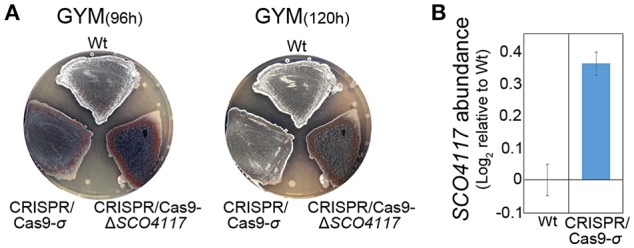
Phenotypical analyses of the CRISPR/Cas9-σ mutant. **(A)** Macroscopic view of sporulation (gray color) in the wild-type strain, the CRISPR/Cas9-σ mutant and the CRISPR/Cas9-Δ*SCO4117* mutant growing in GYM plates. **(B)** *SCO4117* transcript abundance in the CRISPR/Cas9-σ mutant compared to the wild-type strain, both grown in GYM plates at 17 h.

A mutant expressing the sigma factor domain from the *SCO4117* ORF was created by CRISPR/Cas9 (CRISPR/Cas9-σ mutant) (Figure 1). The CRISPR/Cas9-σ mutant shows a delayed actinorhodin production and lower CDA production than the wild-type strain (Supplementary Figure 2). Sporulation is also delayed, but it is faster than in the CRISPR/Cas9-Δ*SCO4117* knockout mutant (Figure 4A). Interestingly, the expression of *SCO4117* is slightly up-regulated (1.3-fold) in the CRISPR/Cas9-σ mutant compared to the wild strain (Figure 4B), suggesting that the sigma factor domain regulates the expression of its own gene.

### Comparison of Δ*SCO4117* and *S. coelicolor* wild-type transcriptomes

**Figure 5:**
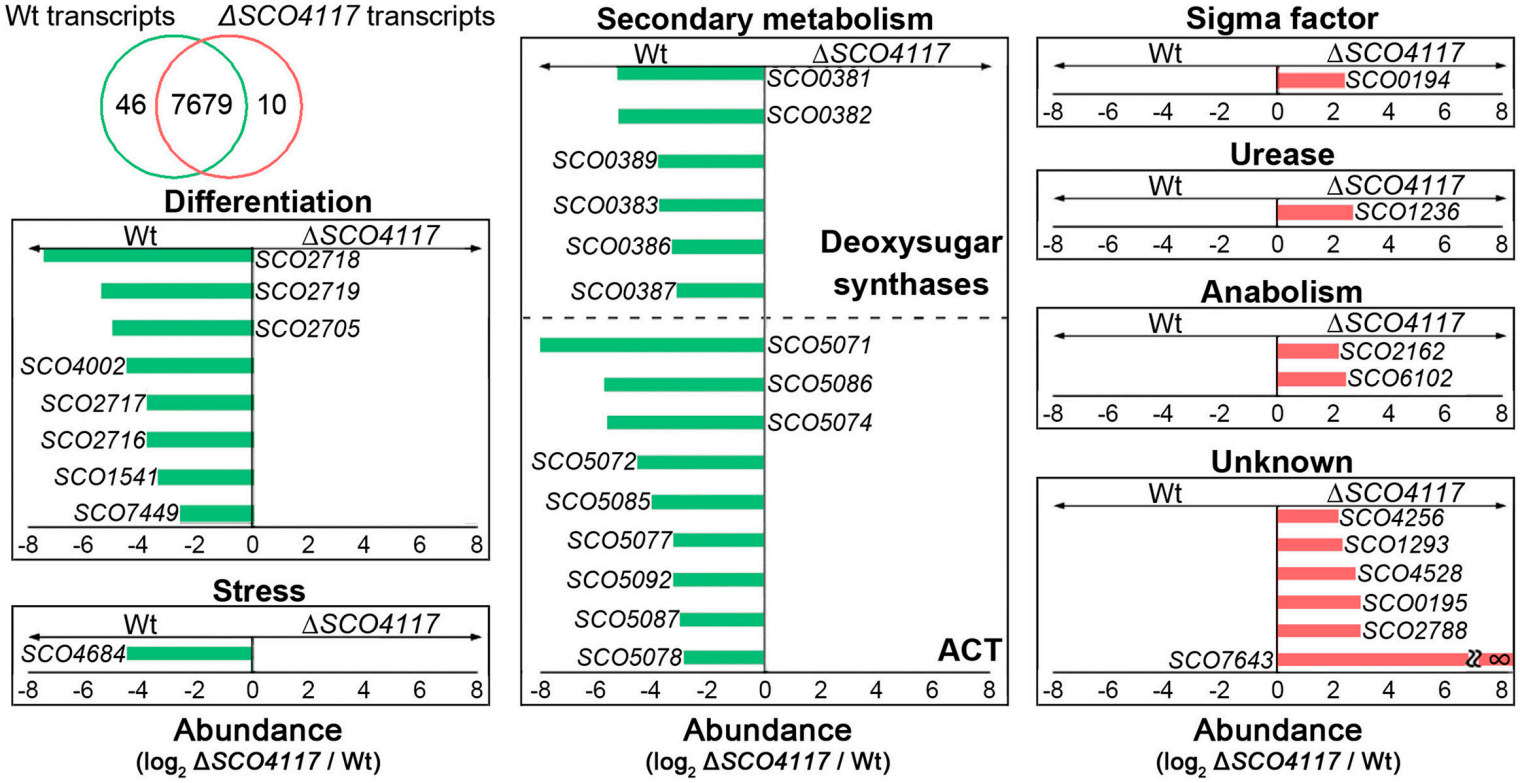
Transcriptomic analysis of the Δ*SCO4117* mutant and the *S. coelicolor* wild-type strain. Δ*SCO4117* mutant vs. *S. coelicolor* transcriptome at 44 h on solid GYM cultures. Venn diagram shows transcripts with significant up-regulation (*p*-value < 0.03) in the wild-type strain (46 transcripts) or the Δ*SCO4117* mutant (10 transcripts) and transcripts without significant variations (7,679 transcripts). Histograms show the abundance of the transcripts with significant variations (*p*-value < 0.03) discussed in the text. Abundance values (average from two biological replicates) are shown. Green bars indicate transcripts up-regulated in the *S. coelicolor* wild-type strain. Red bars indicate transcripts up-regulated in the *SCO4117* mutant.

The differences between the Δ*SCO4117* mutant and the wild-type transcriptomes were analyzed at 44 h on solid GYM cultures, the developmental conditions preceding the differences observed in sporulation (Figures 2E,F). Fifty-six genes showed altered expression in the Δ*SCO4117* mutant compared to the wild-type strain (Supplementary Table 1). As expected, the *SCO4117* transcript was absent in the Δ*SCO4117* knockout (Supplementary Table 1). The expression of the *SCO4117* neighbor genes (*SCO4116* and *SCO4118*) was not significantly affected (Supplementary Table 2). Particularly interesting is that 46 of 56 transcripts, 82% of the total, are down-regulated in the Δ*SCO4117* mutant compared to the wild strain (green bars in Figure 5, Supplementary Table 1), suggesting a pleiotropic activator effect of the SCO4117 ECF which is absent in the knockout mutant. 7,679 transcripts did not show significant variations (*p*-values higher than 0.03) (Figure 5).

Several key developmental and physiological genes were differentially expressed in the Δ*SCO4117* mutant compared to the wild-type strain (Figure 5; Table 2). The expression of genes involved in secondary metabolism (deoxysugar synthases and actinorhodin biosynthetic genes) was highly down-regulated (up to 0.003, i.e., up-regulated 333-fold in the wild-type strain). The expression of genes involved in aerial mycelium formation and sporulation (*chaplins, rodlins, nepA, ssgB, SCO7449*) was also highly down-regulated in the mutant (up to 0.005, i.e., up-regulated up to 200-fold in the wild-type strain) (Figure 5; Table 2). *SCO4684* (encoding the ScoF3 cold shock protein) was down-regulated in the mutant. The other 22 transcripts down-regulated in the Δ*SCO4117* mutant, included, in addition to *SCO4117*, transcripts of genes encoding enzymes, stress and secreted proteins, as well as uncharacterized proteins (Supplementary Table 1). The 10 transcripts up-regulated in the Δ*SCO4117* mutant included: *SCO0194*, encoding a putative sigma factor; *SCO2162*, encoding a quinolinate synthetase; *SCO6102*, encoding a nitrite/sulphite reductase; and 6 transcripts from uncharacterized genes (Figure 5; Table 2).

**Table 2:** Abundance values of transcripts showing significant variations (*p*-value < 0.03) between the Δ*SCO4117* mutant and the *S. coelicolor* M145 wild-type strain quantified in MM solid cultures (44 h), and grouped into functional categories.

**Function**		**SCO no°**	**Gene**	**Log_2_ fold-change (Δ*SCO4117*/Wt)**	**Fold-change (Δ*SCO4117*/Wt)**
Secondary metabolism	Deoxysugar synthases	*SCO0381*	Glycosyl transferase	−5.2	0.03
		*SCO0382*	Dehydrogenase	−5.2	0.03
		*SCO0383*	Glycosyl transferase	−3.8	0.07
		*SCO0386*	Asparagine synthetase	−3.3	0.1
		*SCO0387*	Oxidoreductase	−3.1	0.1
		*SCO0389*	Lipoprotein	−3.8	0.07
	ACT	*SCO5071*	Dehydrogenase	−8	0.003
		*SCO5072*	Dehydrogenase	−4.5	0.04
		*SCO5074*	Dehydratase	−5.6	0.02
		*SCO5077*	*actVA2*	−3.3	0.1
		*SCO5078*	*actVA3*	−2.8	0.1
		*SCO5085*	*actII-4*	−4	0.06
		*SCO5086*	*actIII*	−5.7	0.02
		*SCO5087*	*actIORF1*	−3	0.1
		*SCO5092*	*actVB*	−3	0.1
Differentiation		*SCO1541*	*ssgB*	−3.4	0.09
		*SCO2705*	*chpF*	−5.1	0.03
		*SCO2716*	*chpA*	−3.8	0.07
		*SCO2717*	*chpD*	−3.8	0.07
		*SCO2718*	*rdlA*	−7.5	0.005
		*SCO2719*	*rdlB*	−5.5	0.02
		*SCO4002*	*nepA*	−4.5	0.04
		*SCO7449*	Spore pigmentation^a^	−2.6	0.2
Stress		*SCO4684*	*scoF3*	−4.5	0.05
Regulators		*SCO0194*	Sigma factor	2.4	5.2
Catabolic enzymes		*SCO1236*	Urease	2.7	6.5
Anabolic enzymes		*SCO2162*	Quinolinate synthetase	2.2	4.6
		*SCO6102*	Nitrite/sulphite reductase	2.5	5.7
Unknown		*SCO4256*	Hypothetical protein	2.2	4.6
		*SCO1293*	Putative acetyltransferase	2.3	4.9
		*SCO4258*	Putative hydrolytic protein	2.8	6.9
		*SCO0195*	Putative lipoprotein	2.9	7.5
		*SCO2788*	Hypothetical protein	2.9	7.5
		*SCO7643*	Hypothetical protein	∞^b^	∞^b^

*The genes discussed in the text are indicated*.

a *Secreted protein related to spore pigmentation (Salerno et al., 2013)*.

b *Transcripts not detected in the wild-type strain*.

### SCO4117 DNA motif bound

**Figure 6:**
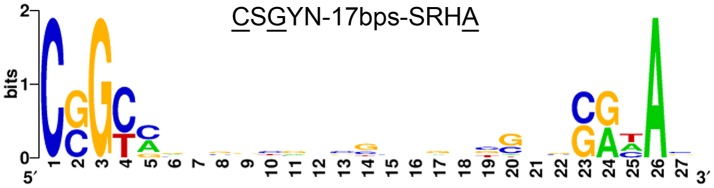
SCO4117 putative DNA motif bound. Putative SCO4117 motif bound logo (“CSGYN-17bps-SRHA”) identified in the promoter region of 29 genes showing affected transcription in the SCO4117 mutant (Supplementary Table 1). The logo was created using Weblogo (Crooks et al., 2004).

The 46 genes encoding transcripts down-regulated in the Δ*SCO4117* mutant (Figure 5) (Supplementary Table 1) might harbor SCO4117 motif bounds in their promoters. These genes were grouped into 33 putative operons (Supplementary Table 1) whose promoter regions were analyzed (see Materials and Methods). Twenty-nine promoters harbored the putative “CSGYN-17bps-SRHA” SCO4117 motif bound at their promoter regions (Figure 6) (Supplementary Table 1).

## Discussion

The study of the *Streptomyces coelicolor SCO4117* knockout mutant revealed that the *SCO4117* ECF sigma factor is a pleiotropic activator of antibiotic production (actinorhodin and CDA) in solid and liquid cultures (Figures 2B–D). Aerial mycelium differentiation and sporulation are also enhanced by SCO4117 in solid sporulating cultures (Figures 2E,F). The effect of SCO4117 activating the expression of secondary metabolism and differentiation was corroborated by transcriptomics. The expression of secondary metabolism (deoxysugar synthases, actinorhodin) and differentiation genes (*bld, rdl, chp, nepA, ssgB*) was dramatically reduced (up to 300-fold) in the knockout strain compared to the *S. coelicolor* wild strain. The effect of *SCO4117* in secondary metabolism was not universal, since the expression of some secondary metabolite genes (for instance the undecylprodigiosin genes) did not appear to be affected (Supplementary Table 2).

Gene *SCO4117* encodes a multidomain ECF sigma factor belonging to the ECF52 family (Figure 1). A putative SCO4117 motif bound (“CSGYN-17bps-SRHA”) was identified (Figure 6). This motif differs from the theoretical ECF52 sigma factor promoter signature predicted by Pinto and Mascher (2016). Both motif bounds are theoretical, but the promoter signature identified in this work was present in the promoter regions of 29 putative operons down-regulated in the *SCO4117* knockout mutant. Further experimentation will be necessary to unequivocally identify the ECF52 sigma factor promoter signature. Unfortunately, our attempts to overproduce the SCO4117 protein to study its interaction with the identified motif, were unsuccessful (data not shown).

*SCO4117* gene expression has a complex regulation. Two promoters were identified controlling the expression of this gene (Jeong et al., 2016), one of them harboring the putative SCO4117 motif bound identified in this work. However, further uncharacterized regulation should exist, as the two promoters together with the *SCO4117* ORF, did not complement the wild-type phenotype (Figure 2G), or the *SCO4117* gene expression (Figure 2H), in the *SCO4117* knockout. Polar effects in the expression of the *SCO4117* neighbor genes due to the apramycin gene insertion, were discarded, because the expression of the *SCO4116* and *SCO4118* genes does not show significant variations in the Δ*SCO4117* compared to the wild strain (Supplementary Table 2) and because the same phenotypes were observed in the CRISPR/Cas9-Δ*SCO4117* mutant and in the insertional Δ*SCO4117* knockout (Figure 3). Our results, might indicate the existence of unknown promoters controlling the expression of *SCO4117*, perhaps a cotranscription of the *atrA* and *SCO4117* genes from the *atrA* promoter. However, Uguru et al. (2005) described a putative transcription terminator between *SCO4118* and *SCO4117*. They also demonstrated that the *atrA* knockout phenotype (severe reduction in the amount of actinorhodin production) is complemented by a DNA fragment containing *atrA*, but lacking *SCO4117* (Uguru et al., 2005). The putative SCO4117 motif bound found in one of the *SCO4117* promoters (Figure 6) (Supplementary Table 1), suggests a positive autoregulation of *SCO4117* gene expression. This autoregulation was observed in a strain expressing a truncated version of SCO4117 lacking the zinc-finger domain, the transmembrane region and the carbohydrate binding module, in which the expression of *SCO4117* is activated (Figure 4B). Positive gene expression autoregulation is common in ECF sigma factors, allowing the amplification of the activation signal (Helmann, 2002; Pinto and Mascher, 2016). Further work will be necessary to fully understand the regulation of the *SCO4117* gene transcription.

The developmental effect of the expression of a truncated version of the *SCO4117* gene encoding the σ factor domain in the CRISPR/Cas9-σ mutant (delay in differentiation/sporulation) was lesser than in the absence of the whole gene in the Δ*SCO4117* mutant (Figure 4), indicating that the σ factor domain itself can modulate development. These results suggest a complex post-translational regulation of the SCO4117 activity which might involve the excision of the ECF sigma factor domain from the membrane by an uncharacterized protease. In fact, Pinto and Mascher (2016) proposed this kind of regulation for ECF52 sigma factors: the sigma factor domain might be activated by the carbohydrate-binding module, leading to a proteolytic cascade or conformational changes that inactivate the zinc-finger domain to ultimately release an active form of the ECF sigma factor. Interestingly, in a previous study, we discovered that SCO4117 is phosphorylated at Ser 15 and Thr 231, suggesting a putative regulation of the SCO4117 activity by phosphorylation (Manteca et al., 2011). Further work will be necessary to fully understand the post-translational regulation of the SCO4117 activity.

Overall, in this work, we demonstrated pleiotropic effects on the regulation of secondary metabolism and differentiation of *SCO4117*, the first member of the ECF52 family characterized. Gene *SCO4117* is a conserved gene overexpressed during substrate and aerial mycelium stages, with complex regulation at the transcriptional and post-translational levels.

## Author contributions

ML-G, PY, NG-Q, and BR performed the experiments. ML-G and AM planned the experiments and wrote the manuscript.

### Conflict of interest statement

The authors declare that the research was conducted in the absence of any commercial or financial relationships that could be construed as a potential conflict of interest.

## Abbreviations

ECF: extracytoplasmic function
ASF: anti-sigma factor
CDA: calcium dependent antibiotic
PCD: programmed cell death
ORF: open reading frame
NGS: next generation sequencing
RT-PCR: reverse transcription PCR.
