# Supplementary figures and images for: The SCO4117 ECF Sigma Factor Pleiotropically Controls Secondary Metabolism and Morphogenesis in Streptomyces coelicolor
Source: Front Microbiol. 2018 Feb 21;9:312. doi: 10.3389/fmicb.2018.00312 (PMC5826349; doi:10.3389/fmicb.2018.00312)

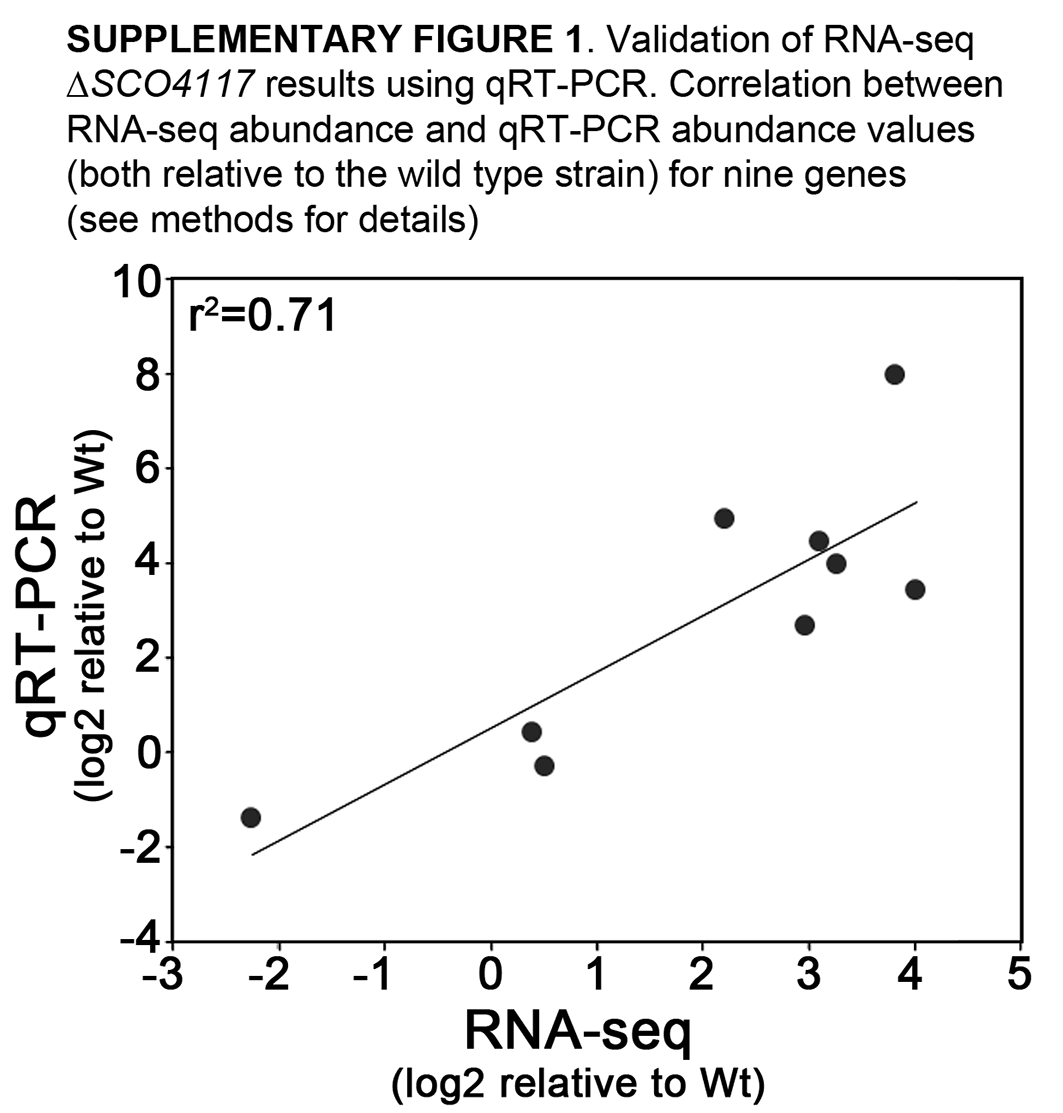

Supplement: Supplementary file 3 [file Image1.TIF]

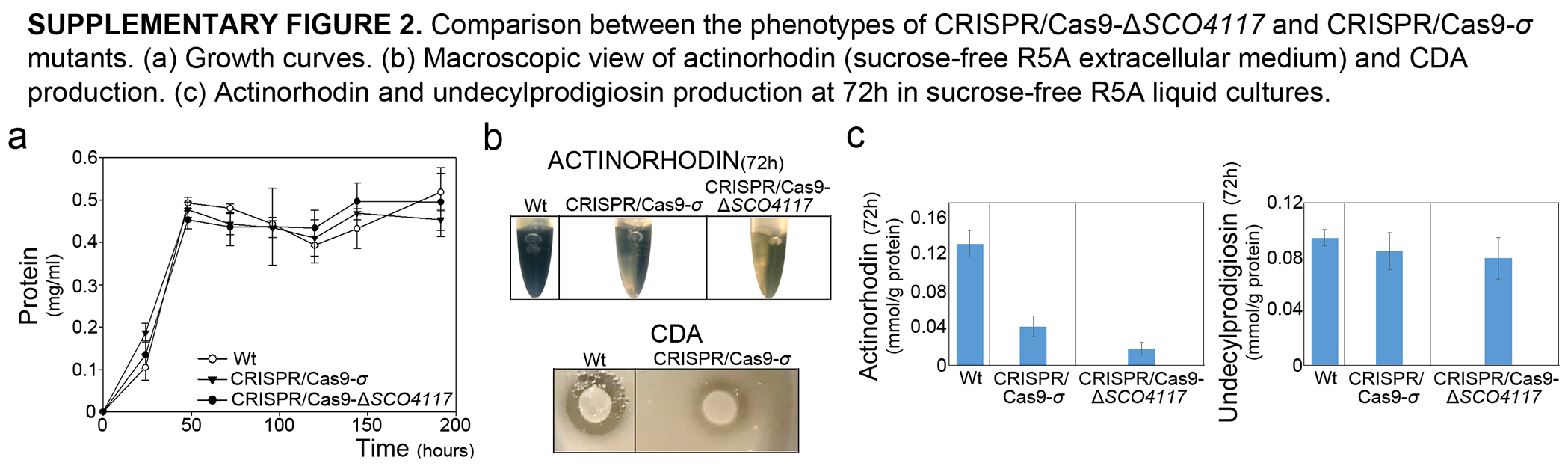

Supplement: Supplementary file 4 [file Image2.TIF]
